# Saudi Arabia Health Systems: Challenging and Future Transformations With Artificial Intelligence

**DOI:** 10.7759/cureus.37826

**Published:** 2023-04-19

**Authors:** Abdullah Saeed, Abdulrahman Bin Saeed, Foton A AlAhmri

**Affiliations:** 1 Action Research, Ministry of Health, Abha, SAU; 2 Public Health, King Abdelaziz University, Khamis Mushait, SAU; 3 Public Health, King Khalid University, Khamis Mushiat, SAU

**Keywords:** hospital management, public health care, artificial intelligence in medicine, kingdom of saudi arabia (ksa), global healthcare systems

## Abstract

The healthcare system in Saudi Arabia is facing several challenges, including an aging population, an increase in chronic diseases, and a shortage of healthcare professionals. To address these challenges, the government is taking proactive steps, including expanding healthcare infrastructure, promoting the use of technology, improving the quality of healthcare services, and emphasizing the importance of preventive healthcare. In addition, the adoption of artificial intelligence (AI) solutions can play a crucial role in transforming the healthcare system by improving efficiency, reducing costs, and enhancing the quality of care. However, the adoption of AI solutions comes with challenges such as the need for high-quality data and the development of regulations and guidelines. The government needs to continue to invest in healthcare and AI solutions to build a more efficient and effective healthcare system that benefits all citizens.

## Editorial

Saudi Arabia's healthcare system faces various issues, including Saudi Arabia having one of the highest incidences of non-communicable diseases (NCDs) such as diabetes and obesity. The treatment of these disorders is costly and places a substantial strain on the healthcare system. Healthcare resources are frequently concentrated in urban regions, leaving rural and distant places with limited access to quality healthcare services. There is a deficiency of healthcare experts, particularly in specialized professions. This shortage might lead to long wait periods and delays in accessing healthcare. Although Saudi Arabia has made progress in implementing technology in healthcare, there is still considerable opportunity for improvement. For instance, telemedicine is not yet commonly utilized, limiting access to healthcare services for persons in remote locations. Some individuals may find it challenging to purchase vital healthcare services in Saudi Arabia due to the country's high healthcare prices. The Saudi Arabian government has started a number of initiatives to improve the healthcare sector in response to these difficulties. These objectives include improving access to healthcare services in rural and isolated locations, boosting the number of healthcare professionals, and investing in technology. In addition, the government has developed programs to encourage healthy lifestyles and avoid noncommunicable diseases such as a sugar tax and physical activity promotion [1].

However, the Saudi Arabian government is taking steps to address these challenges and transform the healthcare system into a more efficient and effective one. One of the key initiatives is the National Transformation Program 2020, which aims to improve the quality of healthcare services, increase access to care, and reduce healthcare costs. The program includes several measures such as increasing the number of healthcare providers, enhancing healthcare infrastructure, and implementing new healthcare technologies. Furthermore, the Vision 2030 plan, which was launched by the Saudi government in 2016, includes a comprehensive strategy for transforming the healthcare system. The plan aims to create a healthcare system that is more patient-centered, innovative, and accessible to all citizens. It also emphasizes the importance of preventive healthcare and health education [2].

The healthcare system in Saudi Arabia is facing several challenges, and the government is taking proactive steps to address these issues. One of the key initiatives that can play a crucial role in transforming the healthcare system is the adoption of artificial intelligence (AI) solutions, which have the potential to revolutionize healthcare by improving efficiency, reducing costs, and enhancing the quality of care. In Saudi Arabia, AI solutions can be used in various areas of healthcare, including diagnosis, treatment, and patient care. An area where AI solutions can make a significant impact is medical diagnoses. AI algorithms can analyze vast amounts of medical data and help healthcare providers diagnose diseases accurately and quickly. This can reduce the burden on healthcare providers and improve the accuracy of diagnosis, leading to better patient outcomes [3].

AI solutions can also be used to develop personalized treatment plans for patients. By analyzing patient data, including genetic information, AI algorithms can identify the most effective treatment options for individual patients. This can lead to better patient outcomes and reduce the likelihood of adverse events. In addition to diagnosis and treatment, AI solutions can also improve patient care. For example, AI-powered chatbots can help patients schedule appointments, access medical information, and receive personalized health advice. This can reduce the workload of healthcare providers and improve patient satisfaction. However, the adoption of AI solutions in healthcare comes with some challenges. One of the primary challenges is the need for high-quality data. AI algorithms require vast amounts of data to be trained effectively, and the quality of data is critical to the accuracy of the algorithms. Another challenge is the need to develop regulations and guidelines for the use of AI solutions in healthcare. The government needs to ensure that AI solutions are used ethically and transparently and that patient privacy is protected [4]. In conclusion, AI solutions have the potential to transform the healthcare system in Saudi Arabia and improve the quality of care for patients. However, adopting AI solutions requires careful planning, investment in technology and infrastructure, and the development of regulations and guidelines.

We hope that the government will continue to invest in AI solutions and work toward building a more efficient and effective healthcare system that benefits all citizens.
